# Application of a Multiplex Ligation-Dependent Probe Amplification-Based Next-Generation Sequencing Approach for the Detection of Pathogenesis of Duchenne Muscular Dystrophy and Spinal Muscular Atrophy Caused by Copy Number Aberrations

**DOI:** 10.1007/s12035-023-03572-9

**Published:** 2023-08-19

**Authors:** Yongchen Yang, Chaoran Xia, Xiaozhen Song, Xiaojun Tang, Xueling Nie, Wuhen Xu, Chengkan Du, Hong Zhang, Peng Luo

**Affiliations:** 1 Department of Laboratory Medicine, Shanghai Children’s Hospital, School of Medicine, Shanghai Jiao Tong University, Building 7, 24, Lane 1400, West Beijing Road, Jing’an, Shanghai, 200040 People’s Republic of China; 2 Zhejiang Shaoxing Topgen Biomedical Technology Co. Ltd. Block B, Building 19, No. 3399 Kangxin Road, Pudong District, Shanghai, 201321 People’s Republic of China; 3 Shanghai Shiji Medical Laboratory Institute, Floor 5, No. 3805, Zhoujiazui Road, Yangpu District, Shanghai, 200093 People’s Republic of China

**Keywords:** Duchenne muscular dystrophy (DMD), Spinal muscular atrophy (SMA), Multiplex ligation-dependent probe amplification (MLPA), Copy number aberrations (CNAs), Next-generation sequencing (NGS)

## Abstract

Both Duchenne muscular dystrophy (DMD; OMIM no. 310200) and spinal muscular atrophy (SMA; OMIM no. 253300/253550/253400/271150) are genetic disorders characterized by progressive muscle degeneration and weakness. Genetic copy number aberrations in the pathogenetic genes DMD and SMN1 lead to alterations in functional proteins, resulting in DMD and SMA, respectively. Multiplex ligation-dependent probe amplification (MLPA) has become a standard method for the detection of common copy number aberrations (CNAs), including DMD and SMN1 deletions, both of which are associated with poor clinical outcomes. However, traditional MLPA assays only accommodate a maximum of 60 MLPA probes per test. To increase the number of targeted sequences in one assay, an MLPA-based next-generation sequencing (NGS) assay has been developed that is based on the standard MLPA procedure, allows high-throughput screening for a large number of fragments and samples by integrating additional indices for detection, and can be analyzed on all Illumina NGS platforms.

**Supplementary Information:**

The online version contains supplementary material available at 10.1007/s12035-023-03572-9.

## Introduction

Both Duchenne muscular dystrophy (DMD) and spinal muscular atrophy (SMA) are genetic disorders that usually present in early childhood and are characterized by progressive muscle degeneration, weakness, and muscle wasting. Newly available targeted treatment options are changing the natural history of SMA, such as the prescription medicine nusinersen. However, few DMD patients survive beyond the third decade because there is no known cure for it, and symptomatic and supportive treatments are the standard [1–3].

DMD is a lethal X-linked recessive genetic disease in humans with an incidence of approximately 1/3500 live births in male infants. Generally, the onset of DMD occurs at a mean age of 2.5 years with a poor prognosis, and the mean age of death is approximately 19 years if proper clinical treatment is not given [2, 3]. Many cases of DMD are the result of mutations in the gene DMD (locus on Xp21.2), which encodes dystrophin. Production of the entire protein stops when a variation is encountered. Approximately one-third of DMD cases are thought to arise because of de novo mutations, with the remaining two-thirds of cases inheriting the mutation from their mothers [3–5]. More than 70% of DMD gene defects result from copy number variations in one or more exons of the DMD gene. A previous Chinese study showed that two regions, ranging from exon 3 to exon 22 and exon 45 to exon 54, have been reported as hot spots for DMD gene deletions, while the most prevalently duplicated regions occur at exons 3–11 and 21–37 [6–8].

SMA is an autosomal recessive disorder with an estimated incidence of 1/6 000 to 1/10 000 [9, 10]. Except for adult-onset SMA (type 4), which has a slow progression, most patients with the other three types of SMA have early onset and a shortened life expectancy. Many cases of SMA are the result of mutations in the gene SMN (locus on 5q13.2), which encodes the survival motor neuron (SMN) protein with 8 exons. The SMA locus contains multiple repetitive and inverted sequences resulting in two highly homologous copies of SMN, namely, SMN1 (telomeric SMN) and SMN2 (centromeric SMN) [11]. Both genes differ by only five nucleotides at the 3′ end of each copy, of which 2 bp are on exons 7 and 8. In a molecular diagnosis of SMA, this small difference can also be used to distinguish SMN1 from SMN2 [12]. Clinical data show that over 95% of SMA patients harbor homozygous deletions of SMN1 or gene conversion at exons 7 or 8 from SMN1 to SMN2, whereas the remaining 5% harbor missense, nonsense, and splice site mutations [13]. A meta-analysis showed that the overall carrier rate of SMA in China was 2.0% [14].

Detection of copy number aberrations (CNAs) or copy number variations (CNVs) in genes associated with disorders is important in genetic diagnostics or analysis and the main methods for CNA estimation include multiplex polymerase chain reaction (multiplex PCR), microarray-based comparative genomic hybridization (aCGH), single-nucleotide polymorphism array (SNP array), CNV-seq, multiplex ligation-dependent probe amplification (MLPA), and hybrid capture-based next-generation sequencing (NGS) [15]. The most prevalent method for detecting DMD caused by CNAs is MLPA, while there are various methods for SMA testing, such as Sanger sequencing, quantitative multiplex PCR of short fluorescent fragments (QMPSF), real-time quantitative PCR (real-time PCR), denaturing high-performance liquid phase chromatography (DHPLC), and PCR restriction fragment length polymorphism (PCR-RFLP).

The MLPA assay has become a widely used assay in laboratories performing genetic tests for molecular diagnosis of several diseases [16, 17]. MLPA can analyze up to 60 DNA sequences in a single reaction and detect CNVs, including small intragenic rearrangements, and it has proven to be a sensitive and accurate tool for rapid CNA estimation at the levels of complete chromosomes or single exons in the whole genome. Since genetic screening by NGS is becoming very popular [18, 19], MLPA technology, running on the Sanger sequencing platform of the ABI 3700 DNA analyzer, incurs a significant operational overhead in genetic screening, necessitating the allocation of specialized equipment, technical knowledge, and human resources for its upkeep. Compared with prior methods, the MLPA-NGS platform is a sequencing and analysis technology that operates on an NGS framework compared with earlier methods. MLPA-NGS achieves high-throughput detection of both sites and samples and is also designed to detect single-nucleotide polymorphisms (SNPs), DNA methylation changes, and relative expression levels of RNA [15, 16]. As NGS continues to mature, many international institutions have developed the MLPA-NGS assay independently and subsequently used it in the development of various detection kits, e.g., Benard-Slagter et al. developed digital MLPA [5]. Meanwhile, we independently developed a similar method of MLPA-based NGS (MLPA-NGS) [20]. Although these methods have different names, they all follow the same principle.

Since both DMD and SMA are common neuromuscular disorders that share various similarities in clinical manifestations, we developed a detection kit for both pathogenic genes based on MLPA-NGS, which includes the following: probe set, primer set, and supporting analysis system for CNA detection of human DMD and SMA. The probe set includes the DMD probe set, the SMN probe set, the internal reference probe set, and the external reference probe set. The primer set is a set of primers with indices that is used for high-throughput sequencing without adding adapters after amplification. The kit can simultaneously analyze all 79 exons of the DMD gene and all 8 exons of the SMN1/2 gene and can report the CNAs of exon 7 and exon 8 in the SMN1/2 gene.

## Materials and Methods

### Materials and Samples

Peripheral blood samples were collected at the Children’s Hospital of Shanghai (Shanghai, China) and Shanghai Shiji Medical Laboratory (Shanghai, China) between January 2017 and January 2018, including ten boys (age, 2–10 years) with clinical manifestations of DMD and one of their mothers, ten SMA samples that were validated by SALSA MLPA Probemix P460 kits (MRC-Holland), and fifteen DMD/SMA CNA negative controls that were not validated by either DMD or SMA kits from MRC-Holland, as shown in Supplementary Table 1. All eleven DMD samples were determined to be DMD positive by SALSA MLPA Probemix P034-B2 DMD-1 and P035-B1 DMD-2 (MRC-Holland). All samples were obtained with written informed consent, and the study was approved by the Research Ethics Committee of the Children’s Hospital of Shanghai. A BD vacutainer PPT™ K_2_EDTA tube (BD Diagnostics, Milan, Italy) was used to collect 3 mL peripheral blood per person. Genomic DNA was isolated from fresh blood samples using a DNA Blood Extraction kit (TIANGEN Biotech (Beijing) Co., Ltd., China) according to the manufacturer’s protocol and was qualified and quantified by a NanoDrop 2000 spectrophotometer (Thermo Fisher Scientific Inc., Waltham, USA) and stored at −20 °C until use.

### MLPA Analysis

MLPA analysis of DMD samples was performed by the SALSA MLPA Probemix P034-B2 DMD-1 and P035-B1 DMD-2 with 116 probes in total (MRC Holland). MLPA analysis of SMA was performed by the SALSA MLPA Probemix P460 SMA with 43 probes (MRC Holland). The test was performed according to the manufacturer’s procedures. Raw data were analyzed with Genemarker v1.85 (SoftGenetics, State College, PA, United States).

### Primer and Probe Set Design and Synthesis

The DMD and SMA probe sets we designed to cover all 79 exons of the DMD gene with 80 targeted sites and all eight exons of the SMN gene, which includes eight pairs of probes in SMN and two pairs of probes on exons 7 and 8 to distinguish SMN1 and SMN2. Furthermore, the probe set also contains three pairs of probes on the X and Y chromosomes for the determination of gender and ten pairs of internal reference probes that detect autosomal chromosomal locations. The probe sequences in both the DMD and SMA probe sets were modified appropriately based on the sequences provided by SALSA. Each pair of probes is seamlessly adjacent to the binding site on the chromosome, consisting of the left (L) probe and the right (R) probe. All L-terminal probes always have a universal primer at the 5′ end, and the sequence starting from the 5′ end is as follows: CCTACACGACGCTCTTCCGATCT. For R-terminal probes, a phosphate group and a universal sequence are added at its 5′ end and 3′ end, respectively, during synthesis, and the sequence starting from the 3′ end is as follows: AGCCCCAAGGGATTCCCAACCTGA. The designed probe sequences without universal primers are shown in Supplementary Table 2. Oligomers were synthesized using DNA phosphoramidite chemistry at Sangon Biotech (Shanghai, China), and oligonucleotides were purified using 8 M denaturing urea polyacrylamide gel electrophoresis (Urea-PAGE). All probes were diluted with distilled water, prepared into a probe mix at a concentration of 2 fmol/μL per probe, and then used as the working solution for the DMD-SMA probe mix. The sequences of upstream and downstream primers used for amplification after ligation in the MLPA-NGS assay were 5′-AATGATACGGCGACCACCGAGATCTACACNNNNNNNNACACTCTTTCCCTACACGACGCTCTTCCGATCT-3′ and 5′-CAAGCAGAAGACGGCATACGAGATNNNNNNNNGTGACTGGAGTTCAGACGTGTGCTCTTCCGATCTCGGGGTTCCCTAAGGGTTGGA-3′, respectively. The N sequences are the index sequences of the upstream and downstream primer. Primers were prepared at a concentration of 20 pmol/μL using distilled water. The sequencing platforms we used only gave the sequence of the upstream index; thus, the samples mixed before sequencing were distinguished by the different indeces of the upstream primers.

### MLPA-NGS Assay

**Fig. 1:**
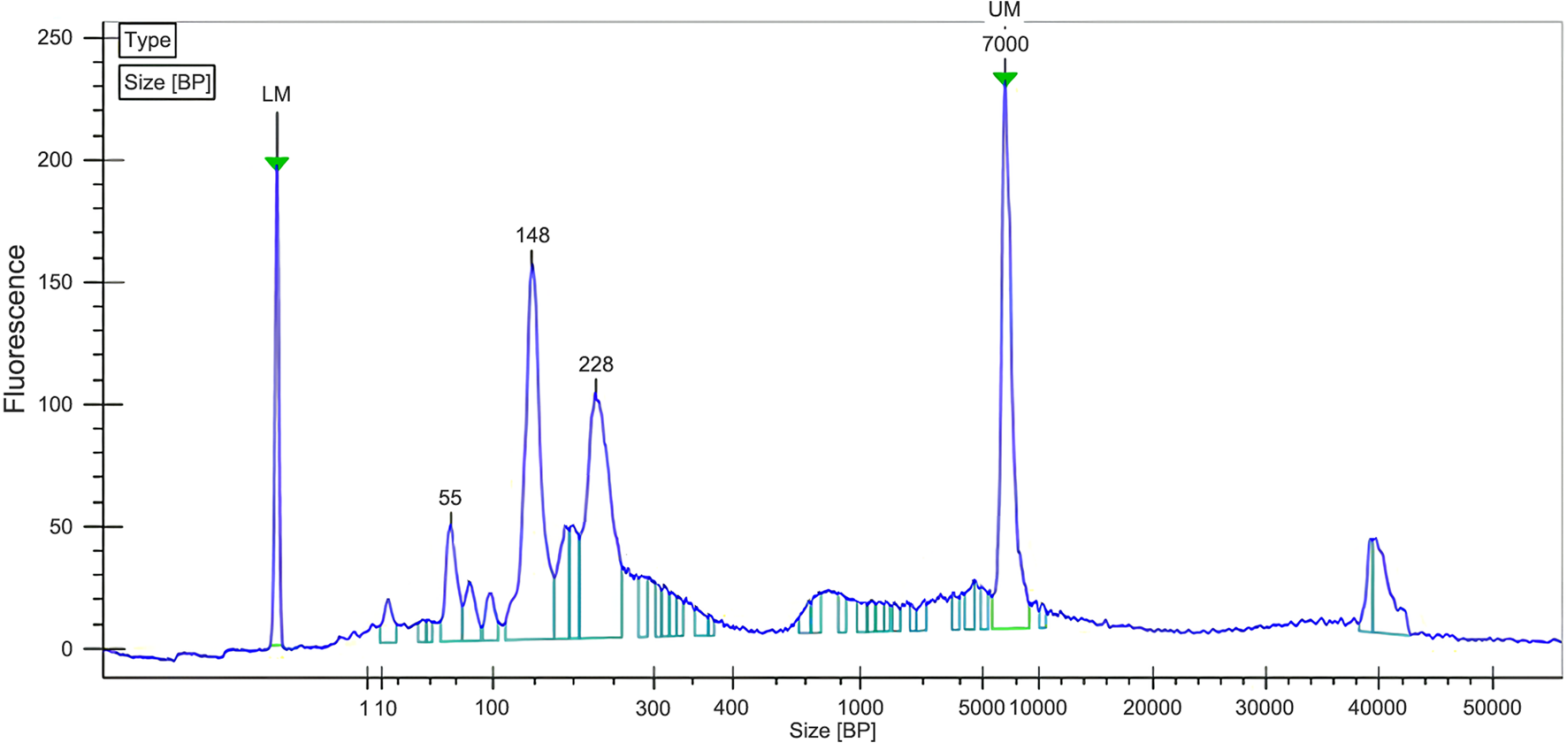
Chart of library quality control by Agilent 2100. LM, lower marker; UM, upper marker. Quality control of the library of the DMD-SMA probe set by Agilent 2100 showed that the main peak was located near 228 bp

The pre-PCR procedure for the MLPA-NGS assay was performed using the MLPA One-Tube MDP-v005 mix (MRC-Holland) according to the manufacturer’s protocol. Initially, 100–500 ng of isolated human genomic DNA dissolved in TE to 5 μL was added into a 0.2-mL PCR tube and then denatured. Then, equal volumes of working solution of the MLPA buffer (MRC Holland) and DMD-SMA probe mix were mixed, and 3 μL was added to each of the PCR tubes. The next day, the ligation solution was prepared for each reaction tube, including 3 μL ligation buffer A, 3 μL ligation buffer B, 1 μL ligase-65 (MRC Holland), and 25 μL dH_2_O. After mixing, the temperature of the PCR instrument was reduced to 54 °C. The lid of the PCR tube on the PCR instrument was opened, and 32 μL of ligase mixture was added, mixed with pipettes adequately, incubated at 54 °C for 15 min, heated at 98 °C for 5 min, and removed from the PCR instrument after incubation at 20 °C. The probe ligation product was then obtained. Finally, the PCR samples were prepared in new PCR tubes. Each PCR tube included 4 μL HS Taq enzyme buffer, 0.25 μL HS Taq enzyme, 3 μL dNTP (Takara, Japan), 1 μL forward primer, 1 μL reverse primer, and 20.75 μL dH2O. Ten microliters of the ligation product was added at room temperature, and then the PCR procedure was performed according to the manufacturer’s protocol. Upstream and downstream primers with barcodes were used for sample identification. Ten microliters of PCR amplification product was taken from each tube and mixed well. The library concentration was initially quantified by a Qubit 2.0 Fluorometer (Thermo Fisher Scientific, Inc.), and the integrity and inserted fragment size of the library were detected by an Agilent 2100 bioanalyzer (Agilent Technologies, Palo Alto, CA) (Fig. 1). If the sample was qualified, forward 40 bp+8 bp sequencing was performed by a NextSeq 550AR gene sequencer (ANOROAD Gene Technology (Beijing) Co., Ltd.); that is, forward sequencing of 40 bp was performed in the probe binding region with gDNA, and the index of the forward primer was sequenced 8 bp. A fastQ file with index information was obtained. To ensure data quality, no less than 0.02 G of raw bases were obtained for each sample.

### Data Analysis

After quality assessment of the exported FASTQ files, total reads were counted in fastQ files, and assignment of the reads to DMD-SMA probe sets and data analysis were performed by a program written in Python with the recommended GRCh38/hg38 reference version for mapping. First, three segments of 10 bp each used as the detective sequence of reads for the 40 bp region were intercepted from the forward to the backward probe in the binding region, as shown in Supplementary Table 2. Each set of sequences was specific for each pair of probes and the corresponding binding sites. The read number generated by each pair of probes for each sample was defined as follows: if a read contained the detective sequence of a pair of probes, the read was considered an analyzable read that was attributed to the probe. The read number corresponded to the area of the peak by capillary electrophoresis of each amplicon in the MLPA assay, reflecting the amount of the original template. Second, the relative amplification of each pair of probes in the DMD-SMA probe sets was obtained by comparing the reads of each pair of probes with the sum of read numbers from the internal reference probes (ATP8A1_E10, IL13_IL4, PKHD1_E61, EYS_E23, CCM2_E2, EYA1_E15, GLDC_E20, ATP7B_E14, BRIP1_E17, and TCF4_I13) located on autosomes. The sex of the sample was determined by the ratio of relative amplification from three pairs of probes located on the Y chromosome (ChrY_SRY_E1, ChrY_UTY_TMSB4Y, ChrY_UTY_E17) to three pairs of probes located on the X chromosome (ChrX_AMOT_E2, ChrX_NR0B1_CXorf21, ChrX_PHEX_E9). A ratio less than 0.1 was considered female, while a ratio greater than 0.5 was considered male. Finally, the relative fragment amplification of each sample was compared with the relative amplification of the corresponding fragments of a control sample. For the DMD gene, if the control was female, the ratio was multiplied by two to obtain the relative copy number of the DMD gene, while if the control was male, the ratio was multiplied by one to obtain the relative copy number of the DMD gene. For the SMN gene, which did not distinguish SMN1 from SMN2, since normal controls contained SMN1 and SMN2, both of which are located on autosomes, the ratio multiplied by four was the relative copy number of the SMN gene in the inspected sample if normal samples with four copies of the SMN gene were used as the control. To distinguish SMN1 from SMN2, the ratio of the inspected sample to the control was multiplied by two to obtain the relative copy number of the SMN1 or SMN2 gene in the inspected sample. We used a healthy male as the control in this study. For the three X chromosome loci and the three Y chromosome loci used to determine sex, ratios were calculated without comparison with controls. In general, quality control was considered acceptable if the value of the relative copy number fluctuated within approximately 30% of the theoretical value, which is the same criteria as a commercial kit (MRC-Holland).

The test accuracy was assessed by whether each positive or negative sample passed quality control. For reference reads of autosomes in each sample, the average ± SD was used for statistics to analyze amplification uniformity.

### Comparison of MLPA-NGS and MLPA Results

To test the reliability of the MLPA-NGS assay, we compared its results with those of MLPA. For each sample, the consistency of the results between MLPA and MLPA-NGS was compared.

## Results

### Analysis of Reads Based on fastQ Files

Automated capillary electrophoresis for quality control of the library sample was performed on a microchip device (LabChip 7500; Calliper Technologies, Mountain View, CA) that is capable of rapidly sizing small DNA fragments by the Agilent 2100 bioanalyzer (Agilent Technologies, Palo Alto, Calif.). The library created by the DMD-SMA probe set had more small fragments (148 bp or smaller), with the main peak located near 228 bp, compared with the quality control for the whole-exome sequencing of genetic diseases that are commonly used in sequencing companies. These small fragments of approximately 148 bp and smaller segments may have been primers that were not involved in amplification. The length of the amplified product was expected to be 217.8±8.6 bp, which was close to the main peak according to the length of the probe and primer. Subsequent sequencing data from forward single-end runs with a 40-bp sequencing length showed that small fragments did not affect the sequencing results. The reads for each pair of probes in each sample were the analyzable reads, as shown in Supplementary Table 3. The size of each fastQ file, the proportion of analyzable reads, and the average and standard deviation of reads on autosomes from internal reference probes were further analyzed, and the results are shown in Supplementary Table 4. The minimum and maximum sizes of the files were 21.7 MB and 36.8 MB, respectively. The maximum size of the file was less than twice the minimum size, and the file size was relatively symmetrical. The read number was at least 792,942 and at most 1,144,366, which was relatively balanced. The minimum and maximum proportions of analyzable reads were 76.88% and 88.92%, respectively, indicating that most of the reads were available according to our criteria. There was no significant correlation between the available proportion of reads and the file size (correlation coefficient, −0.04). For the internal reference reads located on autosomes, the dispersion coefficient was the ratio of the standard deviation to the average of each sample, and the larger the value, the higher the dispersion degree was. In our study, the minimum and maximum values were 0.264 and 0.364, respectively. Thus, the amplified reads of each fragment were relatively symmetrical.

### Copy Number Analysis of DMD-SMA

The relative amplification status of each pair of DMD-SMA probes was obtained by the ratio of the read number of each DMD and SMA locus in each sample to the average read number of the partial internal reference probe that was distributed on the autosomes in the same sample, as shown in Supplementary Table 5. Then, the relative copy number of each targeted site of each sample was obtained by comparing the relative amplification amount of each sample with that of the control, as shown in Supplementary Table 6.

### Comparison of Results by the MLPA-NGS and MLPA Assays

A total of 36 samples were tested in this study. A total of 21 cases were detected by the DMD kit and the SMA kit using MLPA, of which 11 cases were positive for DMD, and 10 cases harbored heterozygous SMN1 deletions. Another 15 DMD/SMA CNA-negative controls were validated as normal samples by both the DMD kit and the SMA kit using MLPA. All the above samples were tested by the DMD-SMA kit using the MLPA-NGS assay, and the results were consistent with those of MLPA, as shown in Supplementary Table 1.

MLPA-NGS technology may reveal novel mutations spanning different exons when detecting copy number variations in the DMD gene; however, the imprecise breakpoint location of the gene renders it inadequate for detecting such mutations. Likewise, SMN1 copy-number tests, designed for specific single-base mutations, lack the capability to discover novel mutations.

Some samples are further described to illustrate the results.

#### A Case with a Hemizygous Deletion of DMD Exons 30-44

**Fig. 2:**
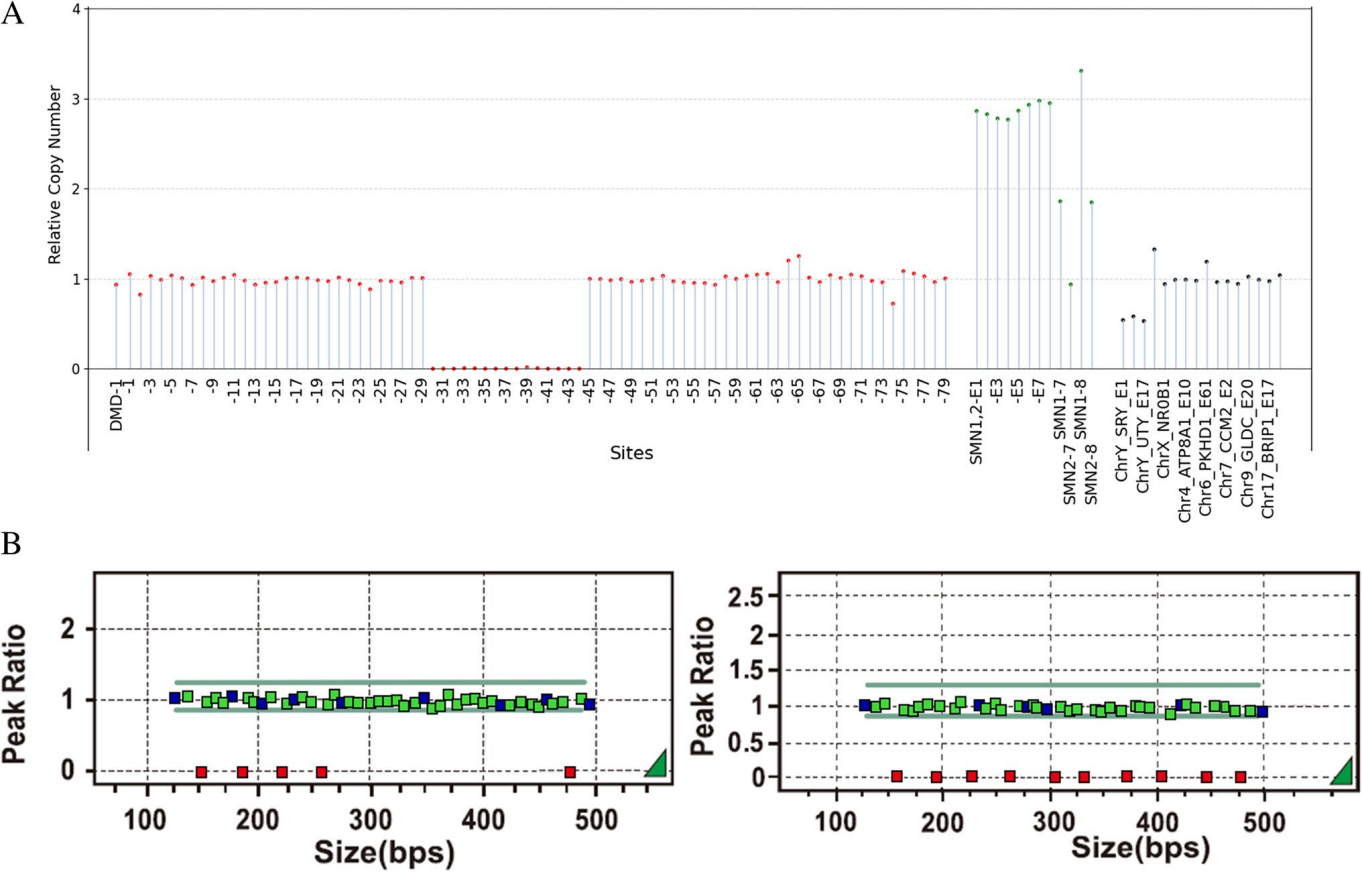
A case with a hemizygous deletion of DMD exons 30–44. **A** The first graph shows the copy number of the DMD gene, including 80 pairs of probes on all 79 exons, arranged by exon number. The second graph shows probes on the SMN gene, including eight pairs of probes in eight exons and four pairs of probes in exon 7 and exon 8 that distinguish SMN1 from SMN2. The third graph includes six pairs of probes distributed on the X and Y chromosomes for sex determination and 10 pairs of probes distributed on autosomes as internal references. The fourth graph is a thumbnail, comparing copy numbers of the DMD/SMA gene, internal references, etc., according to the unified criteria. **B** The results of the MLPA assay. Sites are arranged according to the length of the designed probes. The sequence order was provided by the documentation from MRC Holland (https://www.mrcholland.com/products).

Sample 13 was a male carrying a hemizygous deletion of DMD exons 30-44 according to the MLPA kit (Fig. 2). The results of the DMD-SMA kit showed that the relative copy number of genes on the Y chromosome was greater than 0.5, which was estimated to be a male. Exons 7 and 8 of SMN1 were not deleted, and he harbored a hemizygous deletion of DMD exons 30–44. The relative copy number of the nondeleted sites was within 0.8–1.3, which complied with quality control settings. Although some sites of exons 30–44 had signals and were reflected in the data analysis, the relative copy number of those signals was lower than 0.05, which still complied with quality control settings. Therefore, the test passed the quality control, and the results were consistent with those of the MLPA assay.

#### A Case with a Threefold (3X) Duplication of DMD Exons 3-7

**Fig. 3:**
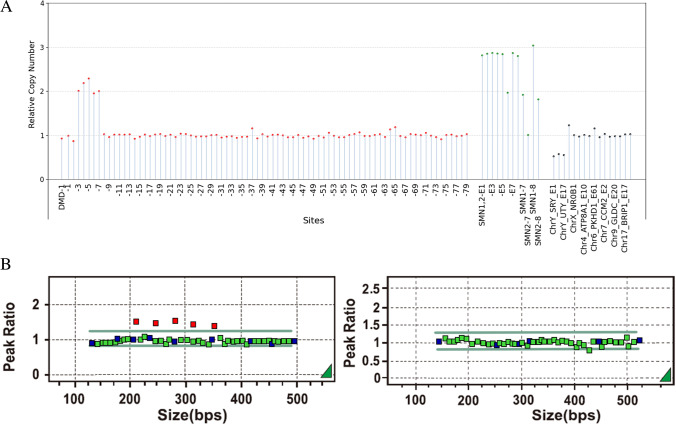
**A**, **B** A case with a threefold (3×) duplication of DMD exons 3–7. Notes please refer to Figure 2

Sample 21 was a female carrying a threefold duplication of exons 3-7 in the DMD gene (Fig. 3). The results of the MLPA-NGS assay also showed that the relative copy number of genes on the Y chromosome was less than 0.1, which was estimated to be female. Exons 7 and 8 of SMN1 were not deleted, and she harbored a threefold (3×) duplication of DMD exons 3–7. The relative copy number of the nonduplicated sites was within 0.8–1.3, which complied with quality control settings. Therefore, the test passed the quality control, and the results were consistent with those of the MLPA assay.

#### A Case of Homozygous Deletion of SMN1 and a Case of Heterozygous Deletion of SMN1

**Fig. 4:**
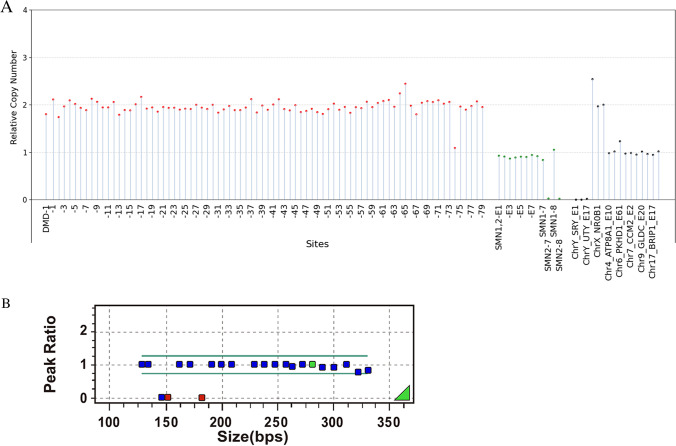
**A**, **B** A case of homozygous deletion of SMN1. Notes please refer to Figure 2

**Fig. 5:**
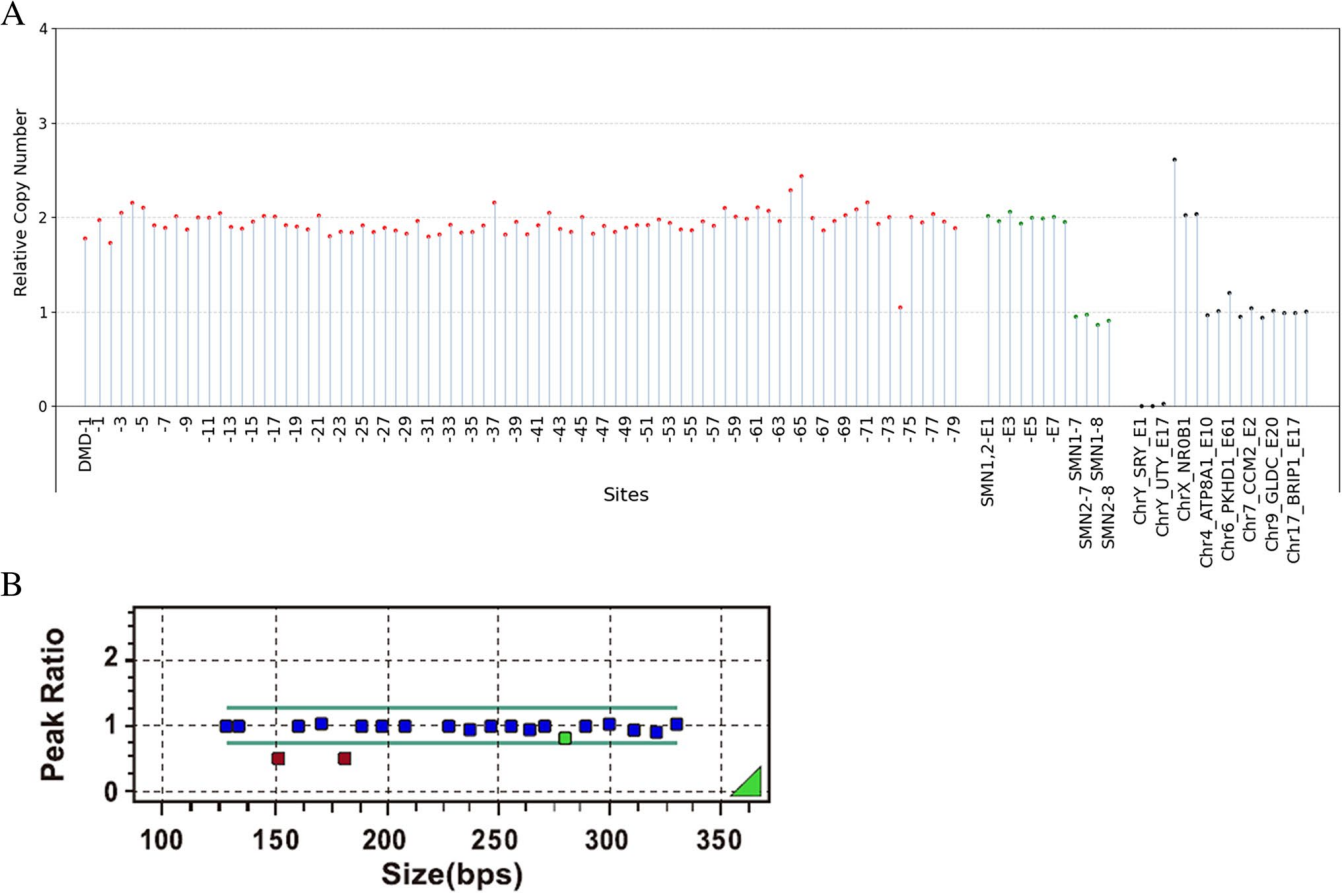
**A**, **B** A case of heterozygous deletion of SMN1. Notes please refer to Figure 2

Sample 2 and sample 1 were female, carrying homozygous and heterozygous deletions of exons 7 and 8 in the SMN1 gene according to the MLPA assay, respectively (Figs. 4 and 5). Exons 1–8 of SMN1 and SMN2 were not distinguished by the MLPA-NGS assay, and the relative copy number was approximately one copy and two copies for Sample 2 and sample 1, respectively, which was consistent with the result of the MLPA assay.

## Discussion

Copy number aberrations (CNAs) have a significant impact on human health and disease. Copy number aberration disorders arise from the dosage imbalance of one or more gene(s), resulting from deletions, duplications, or other genomic rearrangements that lead to the loss or gain of genetic material [1]. The deletion and/or duplication of DNA fragments with a length of more than 1 kb and the complex chromosomal submicroscopic structural variations derived from their combination are the main molecular pathogenesis of genetic disorders and cancer [2].

Multiplex ligation-dependent probe amplification (MLPA) is a variation of PCR that can detect differences in nucleic acid sequences with a small input of DNA for qualitative and semiquantitative analysis of up to 50 genomic DNA sequence differences in one reaction and relies on the process of DNA denaturation, hybridization, ligation, and PCR, and the special probe structure [21–23]. MLPA is mainly used for the detection of CNVs, SNPs, and methylation. In MLPA, the number of targeted sequence templates substantially corresponds to the number of probe pairs ligated. Afterwards, a pair of universal primers with fluorophores are used to amplify the ligated probe sequences in equal proportions at roughly the same rate. Finally, the amplified products are subjected to capillary electrophoresis, the probes are distinguished according to the product length, and the relative amount of the original template is calculated according to the area of the probe peaks. Restricted by the limited diversity of fragment lengths that can be distinguished by capillary electrophoresis, the throughput requirements of molecular detection cannot be met.

A series of improved technologies were developed based on MLPA to expand the throughput of sites to be detected, including MLPA chip [24] and MLPA vs. multiplexed FISH [25]. Although these techniques can expand the throughput of detection sites to a certain extent, the number of sites that can be detected in one reaction is still insufficient sometimes, even at the cost of reduced accuracy. After the development of NGS, many laboratories invariably combined MLPA with NGS to form new assays with different names, such as digital MLPA [26, 27], MLPA-seq [28], and MLPA-NGS [20]. These assays generate PCR amplicons that are quantified using NGS platforms. Sequencing is used to determine the number of reads for each amplicon by each pair of probe sets. More specifically, the universal primer binding sequence in the probe is part of the adapter in the library construction of NGS. After ligation by the probe and the subsequent amplification of the general primer in the process of NGS library construction, NGS onboard can be performed directly. From the fastQ file obtained from sequencing, the relative copy number of each template corresponding to the probe is calculated by read analysis. Several kits have been designed for this method, such as the leukemia detection kit [26, 29]. In this study, we report a copy number variation detection kit based on an MLPA-NGS assay using the DMD and SMN genes as targets. Identification of CNAs, including deletions and duplications, in the DMD and SMN genes at the exon level is performed by the DMD-SMA kit in samples to be tested. Since the MLPA-NGS assay is not based on the length of amplicons but on the nucleic acid sequence itself for differentiation, the sites detected by the DMD-SMA kit at one time are not limited by length diversity. It is not necessary to consider the length of each amplicon but only the specificity of amplification and other issues when designing the probe sets. Up to 1000 DNA sequences can be detected in a single multiplex reaction by the MLPA-NGS assay [26]. Moreover, multiple samples to be tested can be mixed before delivery to the NGS platform because the primers for amplification have barcodes. By allocating 0.02 G of raw bases and 600 reads/MLPA-NGS probe per sample, sequencing costs can be considerably decreased by accommodating up to 50 samples with a single 1 G raw base. In the classic MLPA assay, due to the large number of exons in the DMD gene, SALSA divides it into two kits for the detection of p034 and p035; that is, 49 and 48 detection sites are included together with the reference sites with lengths ranging from 130 to 500 bp, respectively, and adjacent fragments differ in length by 5~12 bp. The MLPA kit used for the detection of the SMN gene is mainly p460, including 23 detection sites in total with internal reference sites, in which the probe lengths range from 131 to 331 bp, and adjacent fragments differ in length by 5 to 20 bp. In addition, each SMN kit also has four DNA quantity control fragments (DQ) with lengths of 61, 67, 72, and 78 bp. Furthermore, these fragments are ligation independent, and their peak heights are inversely proportional to the amount of template DNA.

In our study, the DMD-SMA kit contains 80 sites of 79 exons of the DMD gene (two detection sites are included in the first exon), thus reflecting the copy number at the exon level of the DMD gene. The probe used to distinguish SMN1 from SMN2 is based on the distinction of the last base in the forward probe, but the stability for the detection of CNAs will be reduced. Relatively speaking, the detection stability of CNAs for SMN is much better without discriminating SMN1/2. Therefore, the accuracy of detection for SMN1/2 can be calibrated by the overall CNA calling of the SMN gene. For example, when heterozygous deletions in SMN1 encompassing exon 7 and exon 8 are accompanied by a relative copy number of three for all eight exons of the SMN gene (including SMN1 and SMN2), it suggests that such deletions may have affected the entire SMN1 gene instead of specific exons only. Conversely, finding a heterozygous deletion in exon 7 of SMN1 alongside a threefold amplification of exon 7 in SMN2 and a relative copy number of four for SMN may indicate that the conversion of exon 7 in one SMN1 allele has taken place. Probe sets include internal and external reference points, with internal references on chromosomes 4–7, 9, 17, X, and Y, where well-represented loci are situated (Supplementary Table 2, nos. 185–216). These probes provide the primary reference for determining relative copy numbers of DMD and SMN genes while ensuring robust genome stability by avoiding the easily mutated chromosome 21. Probes on the X and Y chromosomes also determine the sex and the respective copy numbers of these chromosomes. For the external references, the probe was used as a preadded template, and the binding sites of the amplification primers were preconfigured at both ends. The concentration of the external references was extremely low and did not affect the amplification of the normally ligated probe of DNA melting, hybridization, and ligation. In cases without templates added in the reaction system or failure of hybridization or ligation, since only these external reference probes can be used as templates for amplification, their proportions in the final amplification product are extremely high. If there is no amplification product from either external reference probes or other probes, it can be inferred that there is a problem in the amplification process. Compared with MLPA, MLPA-NGS does not need to synthesize probes of more than 100 bp with different lengths of stuffer sequences, negating the difficulty and expense of synthesizing these probes. Therefore, MLPA-NGS allows for easier probe synthesis and kit development than MLPA once the probe sequence is determined. The DMD-SMA kit has the advantages of large probe capacity, reliable detection results, short turnaround time (TAT), and having a convenient operation and low cost, and it is suitable for the detection or screening of suspected cases.

For data analysis, to determine the probe to which each read belonged, some researchers used the minimum edit distance algorithm, and reads were aligned to the template for mutation calling and sequencing depth analysis as in data analysis of genetic tests for disorders [29, 30]. The Levenshtein distance algorithm is undoubtedly a method with lower requirements for computer configuration and faster calculation. However, if an ordinary office computer is used instead of a server, the analysis process using the same algorithm is still extremely slow. In our study, only read attribution needed to be determined without mutation calling. Furthermore, the data analysis method we developed involved designing three feature sequences for each complete probe sequence; there is no essential difference between using fewer or more feature sequences and using regular expressions to find reads that exactly match these featured sequences from the reads of each sample to determine its read number. If the probe for a read has a base error during synthesis or a sequencing error occurs, it will result in reads and featured sequences that are not completely identical, and then alignment will fail. The computation time is proportional to the sequencing volume of the sample. For 0.1 G fastQ sequencing files, the computation time per file was approximately 1–2 min. Compared with the Levenshtein distance algorithm, our data processing method reduced the aligned read number and eliminated the interference of low-quality reads to a certain extent. According to our sequencing data, the smallest proportion of reads available for alignment in each sample was 76.88%. Therefore, although we did not compare our results with those of the Levenshtein distance algorithm, our method for data analysis has sufficient coverage. In terms of operation speed, for the 36 analyzed samples with a total number of reads above 40 M, the analysis of read number was completed within 30 min by an ordinary computer (CPU Core i7-6700@3.40 GHz, memory 16G), which is much faster than using the Levenshtein distance algorithm. In short, the data analysis method for regular expression is suitable for MLPA-NGS data analysis.

The DMD-SMA assay we developed and adopted in this study can analyze the CNAs of a large number of genomic loci of interest with a short TAT of 48 h, which is tolerable and suitable for the detection of genetic disorders. A series of experiments can be performed with low DNA input by a high-throughput sequencing platform at low cost. Data analysis and result interpretation for MLPA-NGS-based assays will be much easier compared to classical assays such as aCGH or whole-genome sequencing because of the appropriate specific approach for CNA detection.

The average number of generated sequencing reads per pair probe by the MLPA-NGS assay exceeded 6000 in our study (Supplementary Table 3), providing sufficient data for precise read quantification and downstream CNA assessment. Reliable CNA detection is practically feasible with only approximately 600 reads/MLPA-NGS probe on average; thus, over 2000 test samples, including reference controls, can be analyzed simultaneously in a single sequencing run using the probe mix containing 108 DMD/SMA-specific and experimental control probes on a mid-flux chip at a maximum of approximately 130 million reads per run (75 bp single read) by a NextSeq 550AR gene sequencer. This allows the rapid screening of large archives for research purposes, although, in diagnostic laboratories where the TAT is often of the utmost importance, flow cells with lower data output might provide the best choice. Using a standard flow cell with v3 chemistry, the 40+8 bp single-read run took approximately 8 h.

As more information about possible SNPs densely distributed in the human genome is discovered, there is always a likelihood of an SNP being present at the ligation site or on the probes, which might influence probe binding and ligation and might thereby cause a false-negative result. SNPs at and around the ligation site can affect probe ligation, while SNPs on the probes can affect the accuracy in the detection of CNAs by MLPA-NGS. SNPs can reduce a probe signal by destabilizing probe-sample DNA binding and can also reduce the success rate of ligation, leading to a decrease in the number of successfully ligated probes at the loci. We also found that SNPs can seriously affect the accuracy of CNV detection, e.g., the existence of a high-frequency SNP on the DMD-74 probe affected the final results (verification results are not shown). Therefore, using the dbSNP146 database to avoid high frequency and validated SNPs and to fine-tune the binding region of probes on the template is of great importance. The length and melting temperature of each probe oligonucleotide can therefore be chosen (in most cases, increased) in such a way as to ensure stable binding to the targeted sites even in the presence of known SNPs. Thus, multiple probes should be designed and added for each target site to prevent impacts based on single-probe alterations that should always be confirmed by a different method.

In conclusion, our study shows that MLPA-NGS-based kits significantly outperform MLPA-based kits in both the flux of sites and samples to be tested. MLPA-NGS is a reliable assay for the detection of CNAs in DMD and SMN compared with multiplex PCR and MLPA due to its advantages of easy experimental design and data analysis, which greatly reduce cost because of high throughput. Assay-designed probe sets will be tested in independent laboratories and assessed for clinical and diagnostic applications in a larger-scale population validation. Our preliminary findings indicate that MLPA-NGS-based kits warrant further consideration as a valuable alternative for genetic testing or screening of individuals newly diagnosed with DMD/SMA or suspected of being carriers.

### Supplementary information

Supplementary Table 1 Blood samples. Supplementary Table 2 Designed probe sequences without universal primers. Supplementary Table 3 Reads for each pair of probes in each sample. Supplementary Table 4 Size of each fastQ file and the proportion of analysable reads. Supplementary Table 5 Read number of each DMD and SMN locus in each sample. Supplementary Table 6 Relative copy number of each targeted site of each sample. Supplementary Table 1. No. 1-160 are the names and sequences of the probes used for each exon of the DMD gene, while No. 161-176 are those of SMN. No. 177-184 are those that can distinguish SMN1 from SMN2. No. 185-196 are those distributed on Y and X chromosomes, and No. 197-216 are internal reference probes that detect autosomal chromosomal locations. (XLSX 163 kb)

## Data Availability

The raw data of NGS can be accessed using accession number PRJNA999185 in SRA of NCBI (https://www.ncbi.nlm.nih.gov/sra).
